# Identification of novel alleles associated with insulin resistance in childhood obesity using pooled-DNA genome-wide association study approach

**DOI:** 10.1038/ijo.2017.293

**Published:** 2018-02-06

**Authors:** P Kotnik, E Knapič, J Kokošar, J Kovač, R Jerala, T Battelino, S Horvat

**Affiliations:** 1 Department of Endocrinology, Diabetes and Metabolic Diseases, University Children’s Hospital, UMC Ljubljana, Ljubljana, Slovenia; 2 Biotechnical Faculty, University of Ljubljana, Domzale, Slovenia; 3 Genialis Inc., Ljubljana, Slovenia; 4 Unit for Special Laboratory Diagnostics, University Children’s Hospital, UMC Ljubljana, Ljubljana, Slovenia; 5 National Institute of Chemistry, Ljubljana, Slovenia; 6 Department of Paediatrics, Faculty of Medicine, University of Ljubljana, Ljubljana, Slovenia

## Abstract

**Background:**

Recently, we witnessed great progress in the discovery of genetic variants associated with obesity and type 2 diabetes (T2D), especially in adults. Much less is known regarding genetic variants associated with insulin resistance (IR). We hypothesized that novel IR genes could be efficiently detected in a population of obese children and adolescents who may not exhibit comorbidities and other confounding factors.

**Objectives:**

This study aimed to determine whether a genome-wide association study (GWAS), using a DNA-pooling approach, could identify novel genes associated with IR.

**Subjects:**

The pooled-DNA GWAS analysis included Slovenian obese children and adolescents with and without IR matched for body mass index, gender and age. A replication study was conducted in another independent cohort with or without IR.

**Methods:**

For the pooled-DNA GWAS, we used HumanOmni5-Quad SNP array (Illumina). Allele frequency distributions were compared with modified *t-*tests and χ^2^-tests and ranked using PLINK. Top single nucleotide polymorphisms (SNPs) were validated using individual genotyping by high-resolution melting analysis and TaqMan assay.

**Results:**

We identified five top-ranking SNPs from the pooled-DNA GWAS analysis within the *ECE1*, *IL1R2*, *GNPDA1*, *HLA-J* and *PYGB* loci. All except SNP rs9261108 (*HLA-J* locus) were confirmed in the validation phase using individual genotyping. The SNP rs2258617 within *PYGB* remained statistically significant for both recessive and additive models in both cohorts and in a merged analysis of both cohorts and present the strongest novel candidate gene for IR.

**Conclusion:**

We report for the first time a pooled-DNA GWAS approach to identify five novel SNPs or genes for IR in a paediatric population. The four loci confirmed in the second validation phase study warrant further studies, especially the strongest SNP rs2258617 within *PYGB*, and provide targets for further basic research of IR mechanisms and for the development of potential new IR and T2D therapies.

## Introduction

Obesity in adults and children is one of the largest worldwide health-care problems. A recent comprehensive analysis of overweight and obesity in 195 countries between 1990 and 2015 has revealed sobering trends in that the rate of increase of obesity in children has been greater than the rate of increase in adults.^1^ Obesity is defined as the accumulation of excess body fat, in addition to the accumulation of adipose tissue in the liver, skeletal muscle and pericardial region, all of which can lead to the development of obesity’s complications.^2^ Insulin resistance (IR) occurs early and is possibly the main mechanism leading to the metabolic complications of obesity, such as type 2 diabetes mellitus (T2D), dyslipidaemia, early atherosclerosis and cardiovascular disease.^3^

Environmental and genetic factors contribute to IR. The main environmental factors are obesity, sedentary behaviour, stress, nutritional factors (such as excessive intake of fructose in liquid form and branched-chain amino acids) and sleep deprivation.^4^ IR is associated with certain well-known human monogenic disorders.^5^ The first single gene mutation responsible for severe IR was discovered in the insulin receptor gene (*INSR*) in 1988.^6, 7^ A comprehensive review of monogenic forms of IR is given by Semple *et al.*,^8^ and we will list here only a few monogenic genes causing severe IR. Directly functionally connected to *INSR* are mutations in *HMGA1*, a transcription factor binding to the *INSR* promotor. Downstream of insulin signalling, mutations were identified in *AKT2* and *AS160*. Lipodystrophy disorders have also resulted in severe IR such as certain alleles in *CAV1*, *PTRF*, *CIDEC* and *BSCL2*. Digenic mutations in *PPARG* and *PPP1R3A* have revealed that a combination of mutations in genes of lipid or carbohydrate metabolism can result in IR. Moreover, several complex syndromes primarily due to severe obesity and hyperphagia have also been associated with severe IR (see also Semple *et al.*^8^). In addition, several genetic variants have been associated with IR in humans using candidate gene and genome-wide association study (GWAS) approaches.^5, 9^ There is a higher incidence of IR with the simultaneous presence of obesity and associated genetic variants.^10^ This implies that genetic predisposition to IR does not necessarily lead to IR, but IR develops as the genetic predisposition interacts with environmental factors, especially excessive body weight. However, it could also be that excessive body weight and IR are both consequences of the same (environmental) exposure.

Several genetic loci have been associated with T2D and are collated in the GWAS catalogue (https://www.ebi.ac.uk/gwas). This catalogue is manually curated in a quality-controlled manner and collects data from the literature of published GWAS assaying at least 100 000 single nucleotide polymorphisms (SNPs) and all SNP–trait associations with *P*-values<1.0 × 10^−5^.^11^ As of 5 October 2017, 179 significant GWAS loci in 21 studies for a term insulin resistance were recorded, but essentially all of these were derived from studies of adult populations. Only three GWAS studies reported associations also in children: an *LEPR* gene variant,^12^ a common variant in the *FTO* gene in a European population,^13^ and several genetic loci in Hispanic children for T2D and IR.^14^

The above-mentioned studies, therefore, refer to the identification of genes associated with IR in fully developed T2D, mostly in adults. Much less is known about the genetic factors important in the early phases of T2D and in the relationship between obesity and IR. One large-scale meta-analysis study in adults identified six previously unknown loci associated with IR, implying that IR loci can function in different ways than T2D genes.^15^ There are no similar studies in children and adolescents specifically designed to identify IR loci. However, the authors of one study demonstrated the cumulative role of several genetic variants linked to T2D (in genes *TCF7L2*, *HHEX*, *SLC30A8*, *WFS1*, *KCNJ11*, *KCNQ1*, *MTNR1B*, *FTO* and *PPARG*) in the development of prediabetes in adolescents. A greater number of variants was associated with a higher likelihood of prediabetes in this population.^16^ In another study, the authors linked genetic variants in genes *TCF7L2*, *IGF2BP2*, *CDKAL1* and *HHEX1A* with oral glucose tolerance test results in adolescents, identifying reduced insulin secretion as an important pathophysiologic factor.^17^ In the study of Xi *et al.*,^18^ two SNPs in or near the *GNPDA2* and *KCTD15* genes were significantly associated with the risk of IR in Chinese children. In a cross-sectional cohort of Greek children and adolescents of European descent, a significant association was detected between *CDKAL1* SNPs and IR. Recently, a candidate gene approach study demonstrated that the Pro12Ala polymorphism in *PPARG* was associated with IR in Mexican children and suggested that this relationship was modified by dyslipidaemia.^19^ Therefore, genetic studies of T2D in adults and epidemiological, as well as non-genetic (such as nutritional and physical activity), studies of IR and T2D in children are numerous, whereas genetic studies specifically aiming to identify novel genes predisposing to IR in children and adolescents are lacking.

GWAS is an approach that allows examination of common genetic variants throughout the genome. Several studies have demonstrated that DNA pooling can detect the most promising candidate SNPs or genes, with considerable savings in time and costs.^20, 21, 22, 23, 24^ Based on a comprehensive review, theoretical calculations and experimental validations comparing classical GWAS studies using individual genotyping to GWAS with DNA pooling suggested that pooling-based GWAS is a much more efficient strategy for identifying genetic associations with diseases or traits.^25^ Perhaps the most important advantage of pooled-DNA GWAS is its efficiency of study design, which requires three orders of magnitude less financial input than GWAS strategies based on individual genotyping in the first phase. In addition, pooled-DNA GWAS can be effectively applied to studies involving smaller populations. Specifically, in rare diseases or in smaller populations such as the Slovenian paediatric population, it is difficult to obtain an appropriately large sample for GWAS using individual genotyping. In the present study, GWAS was performed using DNA pools from cases and controls of obese children and adolescents with and without IR.

## Materials and methods

### Patients

Prior to inclusion in the study, all participants or their legal guardians signed an informed consent. The study protocol was approved by The Slovenian National Medical Ethics Committee (no. 25/10/09). The study included the first cohort of 198 obese children and adolescents managed by the Department of Endocrinology, Diabetes and Metabolism, University Children’s Hospital, Ljubljana, Slovenia for obesity. Characteristics of the subjects are shown in detail in Table 1. For the purposes of the replication of molecular genetic analysis of identified SNPs in an independent population, the second cohort of additional 157 obese children and adolescents, managed by the same department, matched for age, gender status, degree of overweight and IR status to the primary cohort was included. Cohort was divided into an IR+ group (39 boys, 40 girls; mean age=13.9±2.6 years, mean standardized body mass index (BMI-SDS)=2.9±0.5) and IR− group (38 boys, 40 girls; mean age=13.8±2.9 years, mean BMI-SDS=2.8±0.4) using criteria identical to that used for the pooled-DNA GWAS cohort.

Pubertal status of the subjects was determined in both cohorts according to Tanner^26, 27^; it was, however, not used as a matching criterion. Characteristics of the subjects are also shown in detail in Table 1. The two cohorts were comparable regarding the pubertal status ratios. In the original first cohort, there were 17/42 (40%) prepubertal, 23/56 (41%) midpubertal and 59/101 (58%) postpubertal subjects in IR+ group. In the IR− group, there were 25/42 (60%) prepubertal, 33/56 (59) midpubertal and 42/101 (42%) postpubertal subjects. In the replication (second) cohort, there were 8/19 (42%) prepubertal, 30/65 (46%) midpubertal and 39/73 (53%) postpubertal subjects in IR+ group. In the IR− group, there were 11/19 (58%) prepubertal, 35/65 (54%) midpubertal and 34/73 (47%) postpubertal subjects.

Height (cm) and weight (kg) of participants were measured by trained medical staff using validated wall-mounted stadiometers (Quick Medical, Issaquah, WA, USA) and electronic digital scales (Alba, Vojnik, Slovenia). BMI values were calculated as weight (kg)/height^2^ (m^2^). Obesity was defined as a BMI-SDS>2.^28^

### IR assessment

Oral glucose tolerance test was performed in all subjects after an overnight fast. Blood samples were taken before (0 min) and 30, 60 and 120 min after glucose ingestion (1.75 g kg^−1^; maximum, 75 g).^29^ The concentration of glucose was measured using a routine hexokinase-based protocol and an Olympus AU400 Chemistry Analyser (Olympus, Tokyo, Japan). The plasma insulin concentration was assessed with the Immulite 2000 Insulin solid-phase enzyme-labelled chemiluminescent immunometric assay using an Immulite 2000 analyser (Siemens, Berlin, Germany).

IR was determined using the whole-body insulin sensitivity index (WBISI) and homeostatic model assessment—IR (HOMA-IR). WBISI values were calculated using this formula:


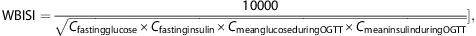


with glucose concentrations in mmol l^−1^ and insulin concentration in pmol l^−1^.^30^ HOMA-IR values were calculated using this formula: 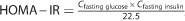, with the glucose and insulin concentrations in the same units as for WBISI.^31^ The WBISI threshold was set at <3 and the HOMA-IR threshold was set at >2.5.

### Genome-wide association study

GWAS was performed on pooled samples of the first cohort of obese children and adolescents to search for genetic variants. Obese adolescents matched for gender, age and BMI-SDS were divided into two groups based on IR status (Table 1). The group of obese children and adolescents with IR (IR+) included 48 boys and 50 girls (mean age=13.5±2.6 years, mean BMI-SDS=3.0±0.5) and the group of obese children and adolescents without IR (IR−) included 50 boys and 50 girls (mean age=12.6±2.9 years, mean BMI-SDS=2.8±0.5). Genomic DNA was isolated from whole blood samples using the FlexiGene DNA Isolation Kit (Qiagen, Hilden, Germany). Before pooling, genomic DNA was diluted to 100 ng l^−1^ to ensure equal representation of each sample. GWAS was conducted by the Beijing Genomics Institute (BGI, Hong Kong, Hong Kong) using SNP-chip Ilumina HumanOmni5-Quad v1.0 (Illumina Inc., San Diego, CA, USA). Each IR+ or IR− pool was assessed three times, generating three replicates per each IR group.

### Data normalization and quality control

To compute the allele frequency estimates from the pooled data, the raw two colour (green/red) bead scores were extracted from the HumanOmni5-Quad (v1.0) array scans. The raw intensity scores, as reported by the Illumina GenomeStudio software (v1.9.4), require calibration before further processing to correct for manufacturing and/or assaying properties that could bias allele frequency estimations.^22, 32, 33^ The green/red ratio tends to systematically differ between different arrays and array strips.^32, 33^ Calibration of the raw intensity data was performed on a strip-by-strip basis by rescaling the red bead score signal to achieve a mean pooling allele frequency (PAF) value of 0.5 for all SNPs on a given strip. The PAF values were computed as the scaled red intensity value divided by the total corrected red plus green intensity value. SNPs for which the mean PAF value was supported by less than four individual beads on a chip (for any given sample) were excluded from further processing. Altogether, on the basis of PAF, we excluded 495 884 SNPs, which represents 11.5% of all SNPs on the microarray. Normalized PAF values were then used for principal component analysis, as implemented in scikit-learn (http://scikit-learn.org), to confirm the clustering of replicate samples. Mean pooling variances and statistical tests of nine comparisons between IR+ and IR− pools are shown in Supplementary Table 1.

### Statistical analysis of GWAS data

A linear model-based approach^22, 32, 33^ was applied using a set of PAF estimates for the IR+ and IR− group comparisons. For each pair of test and control samples, a binominal sampling variance was calculated as V using this formula: 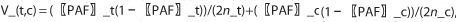 in which PAFt and PAFc were the pooling allele frequencies for the test and control samples, respectively, and *n* was the number of SNPs in a sample. Pooling variance related to the construction of pools from non-identical samples (for example, test and control pools) was estimated as var (epooling-2) according to this formula^33^: 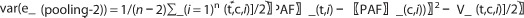. Pooling variance was calculated for each case–control pair of technical replicates separately and then averaged across all comparisons. The estimated pooling variances were used in χ^2^ test as previously described^22^: 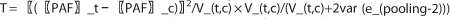. Prioritization of SNPs that differed significantly between groups was performed using a modification of the sliding window method, as described previously.^5^

### Individual genotyping and replication

GWAS results were evaluated by two independent genotyping assays, the high-resolution melting (HRM) analysis and TaqMan test. HRM analysis was performed using MeltDoctor master mix (ThermoFisher Scientific, Waltham, MA, USA) and appropriate oligonucleotide primers (Eurofins Scientific, Luxembourg, Luxembourg) on a 7500 Fast Real-Time PCR System (ThermoFisher). For segment sequencing, a 3500 Genetic Analyser (ThermoFisher) was used. Genotyping using TaqMan assays (ThermoFisher) was also conducted on a 7500 Fast Real-Time PCR System.

### Statistical analysis of SNP genotyping data

Fisher’s exact test for 2 × 2 contingency tables was used to analyse data obtained from SNP genotyping. Online tool VassarStats (Vassar College, Poughkeepsie, NY, USA) enables calculation of *P*-values, odds ratios (ORs) and 95% confidence intervals (95% CIs). Based on these calculations, the allele distribution between two cohorts could be evaluated. The *post hoc* statistical power (1−*β*) of Fisher’s exact test for an *α* of 0.05 was calculated with the G*Power calculator v3.1.^34^ A *P*-value<0.05 and statistical power >50% and a *P*-value<0.05 and statistical power >80% were criteria to include SNP in further analysis. Following the Cochran–Armitage trend test, the combined set of *P*-values by recessive, dominant and additive model were analysed for false discovery rate (FDR) using the two-stage Benjamini, Krieger and Yekutieli FDR procedure.^35^ The *q*-value was optimized in such a way that a set of »discoveries« did not include any potential false positive result (*q*=0,11), consequently only the *P*-values below a threshold of 0.0239 were considered as statistically significant results.

## Results

### Genome-wide association analysis of pooled DNA for identification of novel IR loci in paediatric Slovenian population

We employed a pooled-sample GWAS instead of GWAS on individual DNAs as a cost-effective and feasible method for analysing smaller populations to reduce genetic variability. The underlying hypothesis was that the IR+ DNA pool (participants with IR) would contain more susceptibility alleles for IR than the IR− DNA pool (participants without IR), which could be detected as a difference in allele frequencies or in relative allele signal intensities.

**Figure 1:**
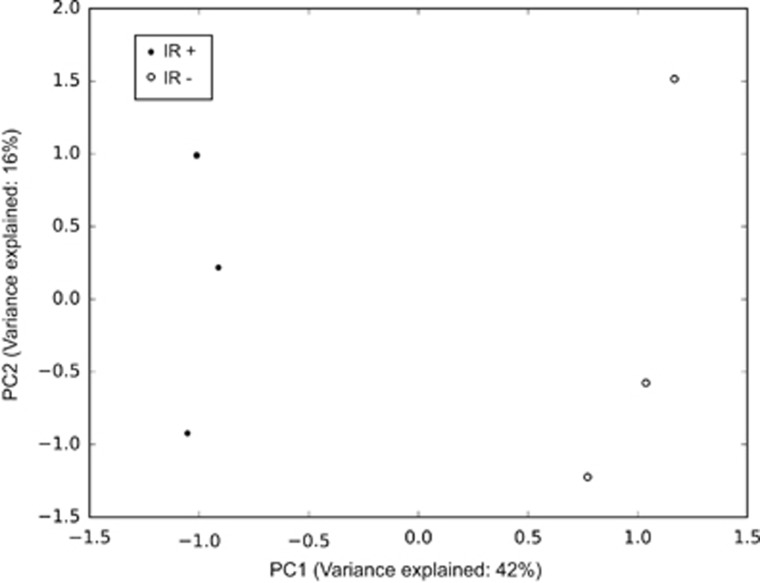
Principal component (PC) analysis plot of distribution of three IR+ pools and three IR− pools. The amount of variance explained by the first two component, PC1 and PC2, is 42% and 16%, respectively. As the percentage of explained variance in the PC2 compared with the PC1 component is low, the spread along the *y* axis alone does not indicate the high within-group sample variability (that is, low technical variance).As IR+ and IR− clusters are far apart, this indicates true global genetic differences (biological variance) between the groups.

In the first step, allele frequency estimates were computed from the raw pooled data of the HumanOmni5-Quad (v1.0) SNP array scans. Following extensive calibration, the mean PAF was calculated and the normalized PAF values were employed in a principal component analysis. Figure 1 shows the principal component analysis plot of the distribution of three IR+ pools and three IR− pools. The three replicates within each group are highly correlated, suggesting low technical variability and, hence, validity of the experimental procedures. The amount of variance explained by the first two component, PC1 and PC2, is 42% and 16%, respectively. Given this relatively low amount of explained variance in the PC2 compared with the PC1 component, the spread along the *y* axis alone is not indicative of the high within-group sample variability. IR+ and IR− clusters are far apart indicating a low correlation between the two groups and, therefore the existence of true global genetic differences (biological variance).

Following computation of the pooling variance, SNPs were ranked by increasing *P*-values derived from the χ^2^ test results. For each SNP, a mean rank in a sliding window of 10 consecutive neighbouring SNPs was calculated (Supplementary Table 1). The sliding window method was used to identify regions that show consistent differences between allele frequencies of SNPs in the case and control groups. The obtained SNP mean rank values were normalized (divided by the number of all tested SNPs) and −log10 transformed before plotting. Supplementary Figure 1 shows Manhattan plots of the top five significant SNPs: rs212540 at Chr1:21266624, rs3218888 at Chr2:102014739, rs252111 at Chr5:142005685, rs9261108 at Chr6:30007810, and rs2258617 at Chr20:25274318. Genomic coordinates for these and other SNPs are displayed along the *x* axis, with the negative logarithm of the SNP’s association *P*-value on the *y* axis. The strongest associations have the smallest *P*-values and hence their negative logarithms are the greatest. The most significant SNPs in the pooled-DNA GWAS analysis were then evaluated in the next step.

### Evaluation of top SNPs from pooled-DNA GWAS analysis on individual DNA samples of the first cohort

The five candidate SNPs (Supplementary Figure 1) identified during GWAS analysis of pooled IR+ and IR− DNA cohorts were selected for re-evaluation on individual DNA samples (first cohort) constituting the DNA pools: 98 children and adolescents from the IR+ group and 100 from the IR− group. To achieve high accuracy and efficiency of genotyping, two different scoring approaches were employed, HRM analysis and TaqMan assay. Table 2A displays the SNP genomic coordinates, associated genes and allelic ratios from the global HapMap project,^36^ European 1000 genomes^37^ and allele frequency ratios from our study. Allelic ratios for all five SNPs from our study are very similar to HapMap and especially to European values, which is expected given the geographical location of the studied Slovenian paediatric population. Table 2B displays minor allelic frequencies in each individual pool with averages and s.e. given separately for IR+, IR− and combined IR+ and IR− populations. Minor allelic frequencies do not deviate much between replicates within IR+ or IR− pools, suggesting low technical variability in our array experiment. However, the mean minor allelic frequencies between the IR+ and IR− pools (Table 2B) become apparent, suggesting that true genetic differences between the IR+ and IR− pools exist.

Following HRM and TaqMan genotyping, we performed statistical analyses for significant differences between the IR+ and IR− groups for each SNP. Results based on HRM identified statistically significant differences between analysed cohorts for a dominant inheritance model for rs212540 and rs252111 (rs212540: *P*-value=0.010, (1−*β*)=0.742, OR=2.315, 95% CI=1.258–4.260; rs252111: *P*-value=0.014, (1−*β*)=0.670, OR=0.421, 95% CI=0.217–0.816) and a recessive inheritance model for rs3218888 and rs2258617 (rs3218888: *P*-value=0.014, (1−*β*)=0.670, OR=2.500, 95% CI=1.216–5.141; rs2258617: *P*-value=0.006, (1−*β*)=0.803, OR=2.795, 95% CI=1.349–5.790). Analysis of rs9261108 did not return statistically significant results and was thus eliminated from further analysis.

The four SNPs exhibiting significant results after HRM genotyping of individuals comprising the GWAS pools were re-genotyped on individual DNA samples using TaqMan probe assays (Table 3) to verify the results obtained by the HRM method. Table 3 presents results of testing the dominant, additive and recessive models of inheritance and provides *P*-values, ORs and 95% CIs. A two-stage Benjamini, Krieger and Yekutieli FDR procedure for FDR was used and *P*-values less than a threshold value of 0.0239 were considered as significant. For SNP rs2258617, a recessive and additive models showed significant associations in the first cohort. SNP rs212540 was significant for a recessive model. For SNP rs252111, we cannot exclude dominant or an additive model though an additive model fits the data best (*P*>0.0062). SNP rs3218888 reached suggestive significance (*P*>0.0397) for a recessive model.

**Figure 2:**
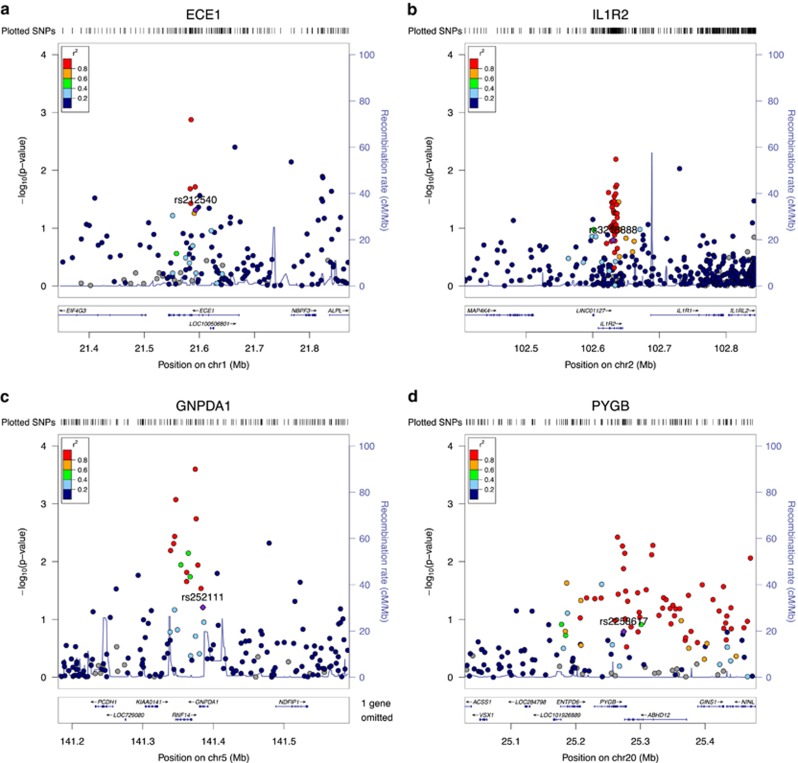
Locus zoom plots for genome-wide significant IR loci that were replicated in individual genotyping of the first cohort. Locus zoom plots are shown for regions with top SNPs within the genes *ECE* on chromosome 1 (a), *IL1RA* on chromosome 2 (b), *GNPDA1* on chromosome 5 (c), and *PYGB* on chromosome 20 (d). Top candidate IR genes are shown on the top of the panel with the most significant SNP indicated within the plot. Closely linked genetic map with the chromosomal physical map are shown on the *x* axis. The unbroken blue line indicates the recombination rate within the region (right *y* axis). Each filled circle represents the log_10_ *P*-value (left *y* axis), with the top SNPs in red, and other SNPs in the vicinity are coloured based on their degree of correlation (*r*^2^) with the top SNP.

Locus zoom plots for these four SNPs are shown in Figure 2. Top significant SNPs lie in a relatively narrow regions on chromosomes 1, 2, 5 and 20. A closer examination of these regions tagged by these SNPs revealed that they were located within genes *ECE1*, *IL1R2*, *GNPDA1* and *PYGB* that have not yet been associated with IR but have roles and functions that can be associated with IR (see Discussion section).

### Replication of top SNPs in a second independent cohort and in a combined analysis of the first and second cohorts

Although our pooled-DNA GWAS results were confirmed by genotype analysis of individual DNA samples from IR+ and IR− cohorts, we aimed at testing the four statistically significant SNPs for replication in an additional independent cohorts of IR+ and IR− obese children and adolescents (Table 1) and in a combined analysis of the first and second cohorts (Table 4). For this replication study, a TaqMan genotyping assay was employed. The participants of the second cohort, who were not related to the members of the pooled-DNA GWAS constituting the first cohort, were divided into an IR+ group (39 boys, 40 girls; mean age=13.9±2.6 years, mean BMI-SDS=2.9±0.5) and an IR− group (38 boys, 40 girls; mean age=13.8±2.9 years, mean BMI-SDS=2.8±0.4) using criteria identical to that used for the pooled-DNA GWAS cohort. Analysis in this second cohort showed suggestive significant differences between groups (Table 4) for rs2258617 located in the *PYGB* gene using the recessive inheritance model (*P*-value=0.039, (1−*β*)=0.533, OR=2.330, 95% CI=1.085–5.003). The other three SNPs failed to return statistically significant results in the second independent cohort (data not shown). Additionally, analysis of all four SNPs was performed on data from a merged first and second cohorts. This combined analysis included 177 obese children and adolescents in the IR+ group (87 boys, 90 girls; mean age=13.9±2.6 years, mean BMI-SDS=3.0±0.5) and 178 obese children and adolescents (88 boys, 90 girls; mean age=13.1±2.9 years, mean BMI-SDS=2.8±0.4) in the IR− group. The SNP rs2258617 within *PYGB* again remained statistically significant for both recessive and additive models and associations were stronger than the association in either of the two cohorts individually (Table 4).

## Discussion

Since its first theoretical studies^20, 38^ and experimental tests,^22, 32^ GWAS analysis using DNA-pooling methodology has proven to be a very time- and cost-effective strategy compared with larger-scale conventional GWAS requiring individual genotyping of the entire study population. Using pooled-DNA GWAS, both known and novel genetic variants have been identified in various diseases or traits.^39, 40^ Of relevance to our study, the pooled-DNA GWAS approach has been successfully used in studies including smaller numbers of participants.^41, 42, 43^ Altogether, the aim of our study was to determine whether GWAS using DNA-pooling methodology could identify genetic variants associated with IR in obese children and adolescents.

Some already known but also novel IR-related loci have been identified in our study following our GWAS-pool analyses. All loci, calculated as sliding window of 10 consecutive neighbouring SNPs, that surpassed statistical significance threshold are shown in Supplementary Table 1. Our single-nucleotide variant rs2237447 (chr7:50640147) maps to the *GRB10* gene very close to the *GRB10* SNP rs10248619 that was significantly associated with fasting glycaemic traits and IR in a GWAS study of Manning *et al.*^15^ Additionally, another risk allele at rs2237457 was shown to be associated with T2D and glucose excursion during oral glucose tolerance test in the Old Order Amish Study.^44^ *GRB10* interacts with insulin receptors and inhibits their signalling^45^ and hence is functionally well connected to the traits in our study. Another single-nucleotide variant rs227070 from our study, is located closely to rs11212617, which was identified as T2D-related locus with both variants mapping to intronic regions of the *ATM* gene.^46^ In this GWAS study examining glycaemic response to metformin in T2D, common variants within the *ATM* gene were reported. *ATM* has been known to cause Ataxia Telangiectasia (A-T; OMIM no. 208900), which is a neurodegenerative disorder but patients also develop marked IR and have increased risk of diabetes.^47^ Additionally, loss-of-function mutation of *Atm* in mice leads to diabetes.^48^ As the third gene, *RNF14*, which was significant in our pooled-sample GWAS analysis, was significant in a GWAS study of associations with amyotrophic lateral sclerosis.^49^ Impaired glucose tolerance in patients with amyotrophic lateral sclerosis has been documented decades ago,^50^ which was confirmed also in several follow-up studies.^51, 52^ Apart from comparing SNP gene-based hits from our list of statistically significant SNPs (Supplementary Table 1), we also compared locations of closely linked regions around our SNPs with studies not reported in the GWAS catalogue. In a recent exome-chip study of genetic variants on diabetes-related metabolic traits,^53^ rs272893 was found significant that is located in *SLC22A4*, a gene found associated with T2D already in previous GWAS studies and closely linked to our significant SNP rs2522052 (Supplementary Table 1). For further detailed analyses on individual analyses in two independent cohorts, we have chosen the five top SNPs because they have not been previously described and because they showed the highest mean rank values. However, the significant candidate SNPs in Supplementary Table 1 present potential new genetic variants to be explored further especially because some of the IR loci detected here in a paediatric population might not show up in similar studies in the adults.

### Candidate SNPs after pooled-DNA GWAS analysis of the first cohort

The pooled-DNA GWAS analysis was performed on pooled samples divided into IR+ and IR− cohorts. Three technical replicates per cohort were used to minimize pooling errors, and the SNP-chip Ilumina HumanOmni5-Quad v1.0 platform was used, which was previously shown to be robust and able to extract maximal available information from pooled DNA.^54^ Additionally, the pooling study design was specifically chosen to reduce further variability not attributable to biological variance of the IR+ group versus IR− group. Individuals constituting the IR+ and IR− groups were carefully selected by matching them for gender, age and BMI-SDS; we consider this to be an important strength of our study. As IR increases physiologically during puberty^5^ and with the degree of overweight,^55^ our matching strategy should have decreased the probability of detecting SNPs associated with the confounding effects of gender, age and degree of obesity instead of IR status.

Five candidate SNPs with the highest statistical significance scores were identified in the pooled-DNA GWAS analysis of the first cohort. None of these five SNPs have been previously associated with IR or any other traits according to the GWAS catalogue database (accessed on 2 February 2017). Four SNPs (rs212540, rs3218888, rs252111 and rs2258617) remained significant after HRM analysis of individual genotypes. These four SNPs were also re-genotyped with the TaqMan assay, as this is more accurate than HRM. Although eventually only one SNP (rs2258617) withstood two further, stringent verification steps, the four significant SNPs after the first-phase pooled-DNA GWAS analysis nevertheless warrant some discussion.

The rs212540 SNP is located in an intron region of the endothelin converting enzyme 1 (*ECE1*) and has been associated with cardiovascular complications of diabetes,^56^ as well as with adult human height^57^ and childhood obesity-related traits in a Hispanic population.^14^ The rs3218888 SNP is located in an intron of the interleukin 1 receptor type 2 (*IL1R2*). This gene is from a family of interleukins that are frequently linked to causes of obesity-associated complications.^58^ The rs252111 SNP that is located in glucosamine-6-phosphate deaminase 1 (*GNPDA1*) has an important housekeeping function in carbohydrate derivative metabolism (Gene Ontology database). In addition, its important paralogue, *GNPDA2*, has been significantly associated with the risk of IR in Chinese children.^18^ SNP rs9261108 is located in the 6p22 region where *HLA-J*, *ZNRD1-AS1* and *RNF39* overlap. *HLA-J* is a pseudogene of *HLA-A*,^59^ a gene associated with Graves’ disease,^60^ an autoimmune-metabolic disorder of the thyroid gland while *RNF39* SNP has recently been associated with non-obstructive coronary artery disease.^61^

Although four out of five significant SNPs in our pooled GWAS analysis continued to be significant in the follow-up validation analysis of individual DNA samples, only SNP rs2258617 within *PYGB* remained significant in the independent second cohort and the merged first and second cohort analysis. The level of association of all four SNPs diminished following the second-phase validation step. Possible explanations for the reduced association in the second cohort may be pool-based or array-based experimental errors or variation in allele frequency because of the relatively small pool sample size. However, we surmise that a major factor may be the relatively small size of the second cohort. Given the high level of statistical significance in the first cohort analysis and the potential functional relevance of the associated genes or regions as discussed above, our results for the aforementioned four SNPs justify further analyses in larger cohorts of Slovenian or other populations.

### rs2258617 (PYGB) is the strongest novel candidate IR SNP

The most robust result of our study was identification of a candidate SNP rs2258617 located in an intron of the glycogen phosphorylase, brain form (*PYGB*) at the 20p11 region. This SNP, as well as neighbouring SNPs, gave high significant values in our pooled-DNA GWAS analysis (Figure 2, Supplementary Table 1). This association was also validated in the same cohort through individual genotyping and confirmed in an independent replication cohort of IR+ and IR− obese children and children and adolescents in Slovenia (Table 4). Statistical analysis for rs2258617 was additionally performed on merged data from the first and second cohorts. Higher statistical values were obtained with this larger population, indicating that our study indeed identified a strong candidate region associated with an increased causal likelihood for IR (Table 4).

SNP rs2258617 resides within the *PYGB* gene,^62^ coding the enzyme that catalyses the rate-determining step in glycogen degradation by releasing glucose-1-phosphate from a terminal alpha-1,4-glycosidic bond. This enzyme thus has a key role in glucose homeostasis. Its activity is regulated allosterically and by reversible phosphorylation.^62^ Mammals have three isozymes of glycogen phosphorylase: liver, muscle, and brain. Liver and muscle isozymes ensure a steady supply of energy to the liver and skeletal muscles, respectively. The brain form is responsible for ensuring glucose supply to the brain, especially under stressful conditions.^63^ Although the name implies specificity for brain tissues, several transcriptome studies clearly demonstrate its expression and possible function in several other tissues, with some tissues (for example, epithelial cells, thyroid, heart, colon) exhibiting even higher expression than in the brain.^64^

In the GWAS catalogue database, no results were found for the rs2258617 SNP, thereby suggesting that we potentially identified a novel candidate gene for IR. However, one GWAS^65^ aimed at identifying genetic variants for serum calcium concentrations found a hit for a closely linked SNP within the *PYGB* gene. The effects of serum calcium levels on insulin release were established decades ago,^66^ and subsequent population studies confirmed that perturbed calcium homeostasis correlates with abnormalities of fasting serum glucose, IR and pancreatic beta-cell function.^67^ Such studies indicate that *PYGB* very likely has functional relevance in IR, possibly through its actions in other tissues besides the brain. Further support for this comes from tissue and developmental stage-specific data collated in the mouse. The mouse studies demonstrate that expression of *Pygb* is high during embryogenesis and in the adult nervous, visceral, endocrine, liver and biliary systems. One other study also found significant differential protein expression of *Pygb* in T2D mice treated with rapamycin for cardiac dysfunction.^68^ Moreover, in an *in vitro* study of pancreatic cancer cells, inhibiting PYGB increased the sensitivity of cells to glucose starvation, partially explaining the manner in which glucose is restricted in tumour cells.^69^ Although the *PYGB* gene has not been comprehensively studied, especially in dedicated analyses of insulin and glucose homeostasis, the above studies that collaterally found associations between *PYGB* and glucose metabolism and IR imply that *PYGB* may act as a pleotropic gene that is not necessarily connected with a brain-specific function, despite its name.

## Conclusions

Using pooled-DNA GWAS analyses, we identified five SNPs and corresponding genes significantly associated with IR in a population of obese children and adolescents: rs212540 (*ECE1*), rs3218888 (*IL1R2*), rs252111 (*GNPDA1*), rs9261108 (*HLA-J*), and rs2258617 (*PYGB*). Significant associations were validated for four SNPs (rs212540, rs3218888, rs252111 and rs2258617) on follow-up analyses of individual DNA samples, whereas rs2258617 (*PYGB*) continued to be significant in an independent cohort and in a merged analysis of the first and second cohorts. To our knowledge, the five SNPs from the pooled-DNA GWAS analysis have not been previously reported in GWAS studies as being associated with IR or related traits. For these five regions, and especially the four that were validated in a replicative individual DNA analyses, it would be of interest to further investigate their possible association with IR in genetic studies of larger cohorts and other functional studies.

The main result of our study is the identification of rs2258617 in the *PYGB* gene as being associated with significant differences in frequencies of alleles between the IR+ and IR− groups. This SNP was significant in the pooled-DNA GWAS analysis, validation analyses of individual genotypes, replication study in an independent cohort and merged analysis of the first and second cohorts. A recessive or additive mode of inheritance was supported by a high OR and low *P*-value. As the HapMap and European population frequencies show very close to intermediate frequencies for the C:T SNP rs2258617, such frequencies are likely expected also in the background (Slovenian) population. In the large first cohort, allele C was much more frequent in the IR− group (60%) than in the IR+ group (48%). This suggests that this SNP is a common and frequent allele in the population, which can potentially serve as an informative diagnostic genetic marker for early detection of IR in obese children and adolescents. Much research remains to be conducted to explore the mechanism by which *PYGB* genetic variants affect IR, which could lead to the development of novel preventative or therapeutic strategies to combat IR. This, in turn, offers the prospect of personalizing treatment based on genotype and opens a route for exploring novel drug treatment opportunities.

In conclusion, we report for the first time a pooled-DNA GWAS analysis of IR and insulin-sensitive obese children and adolescents in the Slovenian population identifying five significant SNPs or genes. Strongest support in validation and replication studies was found for the rs2258617 SNP, suggesting that the *PYGB* gene may be involved in the genetic control of IR and thereby providing a new target for further basic research of the mechanisms underlying IR and for the development of potential new therapies for IR and T2D.

## Figures and Tables

**Table 1:** Cohorts of obese adolescents with (IR+) and without (IR−) IR

	*Cohort 1*		*Cohort 2*		*Whole tested population*	
	*IR+*	*IR−*	*IR+*	*IR−*	*IR+*	*IR−*
*Gender, n*						
Males	48	50	39	38	87	88
Females	50	50	40	40	90	90
						
Age (years)	13.8 (13.3–14.3)	12.6 (12.0–13.1)	13.9 (13.3–14.5)	13.8 (13.2–14.4)	13.8 (13.5–14.2)	13.1 (12.7–13.5)
BMI-SDS (kg m^−2^)	3.03 (2.93–3.14)	2.85 (2.76–2.94)	2.88 (2.77–2.98)	2.82 (2.72–2.92)	2.96 (2.89–3.04)	2.84 (2.77–2.90)
HbA1c (%)	5.20 (5.15–5.26)	5.19 (5.13–5.24)	5.15 (5.10–5.21)	5.13 (5.08–5.19)	5.18 (5.14–5.22)	5.16 (5.12–5.2)
HOMA-IR	4.62 (4.22–5.02)	1.85 (1.70–1.99)	4.57 (4.08–5.06	1.81 (1.63–1.98)	4.60 (4.29–4.91)	1.83 (1.72–1.94)
WBISI	2.23 (2.11–2.36)	5.58 (5.08–6.08)	2.28 (2.15–2.42)	5.93 (5.35–6.51)	2.26 (2.16–2.35)	5.73 (5.36–6.11)
Systolic blood pressure (mm Hg)	128.8 (126.2–131.3)	123.5 (121.0–126.1)	126.5 (123.8–129.2)	124.6 (122.2–127.1)	127.7 (125.9–129.6)	124.0 (122.2–125.8)
Diastolic blood pressure (mm Hg)	68.8 (66.6–71.0)	64.8 (63.0–66.5)	65.7 (63.7–67.7)	64.5 (62.8–66.3)	67.4 (65.9–68.9)	64.7 (63.4–65.9)
Total cholesterol (mmol L^−1^)	4.11 (3.98–4.25)	4.24 (4.09–4.40)	4.26 (4.06–4.46)	3.99 (3.83–4.15)	4.18 (4.06–4.30)	4.13 (4.02–4.24)
LDL (mmol L^−1^)	2.49 (2.37–2.60)	2.57 (2.44–2.71)	2.62 (2.45–2.80)	2.38 (2.24–2.52)	2.55 (2.45–2.65)	2.49 (2.39–2.59)
HDL (mmol L^−1^)	1.11 (1.06–1.16)	1.20 (1.15–1.26)	1.10 (1.04–1.15)	1.17 (1.11–1.22)	1.11 (1.07–1.14)	1.19 (1.15–1.23)
Triglycerides (mmol L^−1^)	1.39 (1.26–1.53)	1.07 (0.97–1.18)	1.31 (1.18–1.44)	1.04 (0.94–1.14)	1.36 (1.26–1.45)	1.06 (0.99–1.13)

Abbreviations: BMI-SDS, standardized body mass index; CI, confidence interval; HbA1c, glycated haemoglobin; HDL, high-density lipoprotein; HOMA-IR, homeostatic model assessment—insulin resistance; LDL, low-density lipoprotein; WBISI, whole-body insulin sensitivity index. Values are represented as mean (95% CI), except for gender where it is presented as number.

**Table 2A:** Top five candidate SNPs from GWAS-pool analysis on individual samples that composed the pools

*SNP*	*Location (GRCh38.p2)*	*Gene*	*Allele ratio (HapMap)*	*MAF (European 1000 Genomes)*	*Mean allele frequencies (our study)*
rs212540	1:21266624	*ECE1*	A:G=0.588:0.412	A:G=0.600/0.400	A:G=0.592/0.408
rs3218888	2:102014739	*IL1R2*	C:T=0.108/0.892	C:T=0.116/0.884	C:T=0.113/0.887
rs252111	5:142005685	*GNPDA1*	C:T=0.195/0.805	C:T=0.178/0.822	C:T=0.129/0.871
rs9261108	6:30007810	*HLA-J*	G:A=0.960/0.040	G:A=0.949/0.051	G:A=0.931/0.069
rs2258617	20: 25274318	*PYGB*	C:T=0.562/0.438	C:T=0.569/0.431	C:T=0.515/0.485

Abbreviations: GWAS, genome-wide association study; MAF, mean allele frequency; SNP, single-nucleotide polymorphism. In Table 2A, we show SNP chromosome location, associated gene, mean allele frequencies (MAFs) from HapMap (35), 1000 genomes from the European population (34) and from our study.

**Table 2B:** Top five candidate SNPs from GWAS-pool analysis on individual samples that composed the pools

*Sample*	*rs212540*	*rs3218888*	*rs252111*	*rs9261108*	*rs2258617*
1. IR+	0.358	0.147	0.922	0.913	0.477
2. IR+	0.352	0.138	0.895	0.874	0.462
3. IR+	0.355	0.160	0.944	0.909	0.493
Average IR+ pool	0.355	0.148	0.920	0.899	0.477
S.e.m.	0.003	0.011	0.025	0.021	0.015
4. IR−	0.476	0.084	0.828	0.964	0.570
5. IR−	0.451	0.085	0.774	0.949	0.541
6. IR−	0.457	0.065	0.865	0.980	0.545
Average IR− pool	0.462	0.078	0.823	0.964	0.552
S.e.m.	0.013	0.011	0.046	0.016	0.016
Mean IR+ and IR−	0.408	0.113	0.871	0.931	0.515
S.e.m.	0.059	0.040	0.063	0.040	0.043

Abbreviations: GWAS, genome-wide association study; IR, insulin resistance; SNP, single-nucleotide polymorphism. In Table 2B, we show frequencies of the B allele in each pool-array experiment with averages and s.e. separately for the pools IR+, IR− and combined IR+ and IR− together. Our frequency data are essentially similar to global mean allele frequency (MAF) and EUR 1K MAF, and the variability between the pools is very low.

**Table 3:** Statistical analysis for each SNP after HRM and TaqMan genotyping of individual DNAs from the first cohort of IR+ and IR− groups

*First cohort*	*rs2258617 (PYGB)*			*rs212540 (ECE1)*			*rs3218888 (IL1R2)*			*rs252111 (GNPDA1)*		
	*C→T*			*A→G*			*C→T*			*C→T*		
	*Recessive*	*Dominant*	*Additive*	*Recessive*	*Dominant*	*Additive*	*Recessive*	*Dominant*	*Additive*	*Recessive*	*Dominant*	*Additive*
OR	2.412	1.495		2.135	1.317		0.4797	NA		NA	0.4484	
95% CI	1.174–4.836	0.7978–2.729		1.177–3.832	0.6379–2.622		0.2437–0.9366	NA		NA	0.2351–0.844	
*P*-value	**0.0168**	0.217	**0.0235**	**0.0164**	0.4745	0.0391	**0.0397**	>0.9999	0.0638	0.1227	**0.0169**	**0.0062**
*q*-Value	0.0907	0.4367	0.1081	0.0907	0.5877	0.1278	0.1278	0.9199	0.1712	0.2822	0.0907	0.0907
1−*b* (*a*=0.05)	0.67	0.24	0.99	0.67	0.11	0.99	0.53	1	0.99	1	0.67	0.99

Abbreviations: CI, confidence interval; HRM, high-resolution melting; NA, not applicable; OR, odds ratio; SNP, single-nucleotide polymorphism. Results of testing the dominant, additive and recessive models of inheritance are shown with *P* values, ORs and 95% CIs. A two-stage Benjamini, Krieger and Yekutieli false discovery rate (FDR) procedure for FDR was used and *P*-values<0.0239 were considered as significant »discoveries« and are indicated in bold text style..

**Table 4:** Statistical analysis for rs2258617 after TaqMan assay performed on original and independent cohorts and merged data from both cohorts

	*First cohort*		*Second cohort*		*Merged cohorts*	
	*IR+*	*IR−*	*IR+*	*IR−*	*IR+*	*IR−*
*Gender*						
Males	48	50	39	38	87	88
Females	50	50	40	40	90	90
						
Age (years)	13.8±2.6	12.6±2.9	13.9±2.6	13.8±2.9	13.9±2.6	13.1±2.9
BMI-SDS (kg m^−2^)	3.0±0.5	2.8±0.5	2.9±0.5	2.8±0.4	3.0±0.5	2.8±0.4

*rs2258617 (PYGB)*									
*C→T*	*Recessive*	*Dominant*	*Additive*	*Recessive*	*Dominant*	*Additive*	*Recessive*	*Dominant*	*Additive*
OR	2.412	1.495		2.33	1.137		2.374	0.8281	
95% CI	1.174–4.836	0.7978–2.729		1.054–4.923	0.5463–2.414		1.397–3.97	0.5185–1.313	
*P*-value	**0.0168**	0.217	**0.0235**	**0.0389**	0.8492	0.2456	**0.001**	0.4725	**0.0133**
*q*-Value	**0.0907**	0.4367	0.1081	0.1278	0.9199	0.4652	0.0322	0.5877	0.0907

Abbreviations: BMI-SDS, standardized body mass index; CI, confidence interval; OR, odds ratio. Results of testing the dominant, additive and recessive models of inheritance are shown with *P*-values, ORs and 95% CIs. A two-stage Benjamini, Krieger and Yekutieli false discovery rate (FDR) procedure for FDR was used and *P*-values<0.0239 were considered as significant »discoveries« and are indicated in bold text style.

## References

[bib1] GBD 2015 Obesity Collaborators. Health effects of overweight and obesity in 195 countries over 25 Years. N Engl J Med 2017; 377: 13–27. 28604169 10.1056/NEJMoa1614362 PMC5477817

[bib2] World Health Organization [Online]. Available from http://www.who.int/dietphysicalactivity/childhood/en/.

[bib3] Switzer NJ, Mangat HS, Karmali S. Current trends in obesity: body composition assessment, weight regulation, and emerging techniques in managing severe obesity. J Interv Gastroenterol 2013; 3: 34–36.

[bib4] Weiss R, Bremer AA, Lustig RH. What is metabolic syndrome, and why are children getting it? Ann NY Acad Sci 2013; 1281: 123–140. 23356701 10.1111/nyas.12030 PMC3715098

[bib5] Semple RK. EJE PRIZE 2015: How does insulin resistance arise, and how does it cause disease? Human genetic lessons. Eur J Endocrinol 2016; 174: R209–R223. 26865583 10.1530/EJE-15-1131

[bib6] Yoshimasa Y, Seino S, Whittaker J, Kakehi T, Kosaki A, Kuzuya H et al. Insulin-resistant diabetes due to a point mutation that prevents insulin proreceptor processing. Science 1988; 240: 784–787. 3283938 10.1126/science.3283938

[bib7] Kadowaki T, Bevins CL, Cama A, Ojamaa K, Marcus-Samuels B, Kadowaki H et al. Two mutant alleles of the insulin receptor gene in a patient with extreme insulin resistance. Science 1988; 240: 787–790. 2834824 10.1126/science.2834824

[bib8] Semple RK, Savage DB, Cochran EK, Gorden P, O’Rahilly S. Genetic syndromes of severe insulin resistance. Endocr Rev 2011; 32: 498–514. 21536711 10.1210/er.2010-0020

[bib9] Kovač J, Šutuš Temovski T, Rozmarič T, Horvat S, Beltram J, Trebušak Podkrajšek K et al. DEPTOR promoter genetic variants and insulin resistance in obese children and adolescents. Pediatr Diabetes 2017; 18: 152–158. 26871578 10.1111/pedi.12371

[bib10] Gerich JE. The genetic basis of type 2 diabetes mellitus: impaired insulin secretion versus impaired insulin sensitivity. Endocr Rev 1998; 19: 491–503. 9715377 10.1210/edrv.19.4.0338

[bib11] Hindorff LA, Sethupathy P, Junkins HA, Ramos EM, Mehta JP, Collins FS et al. Potential etiologic and functional implications of genome-wide association loci for human diseases and traits. Proc Natl Acad Sci USA 2009; 106: 9362–9367. 19474294 10.1073/pnas.0903103106 PMC2687147

[bib12] Go MJ, Hwang JY, Jang HB, Heo L, Park TJ, Lee HJ et al. A genome-wide association study identifies a LEPR gene as a novel predisposing factor for childhood fasting plasma glucose. Genomics 2014; 104: 594–598. 25223902 10.1016/j.ygeno.2014.09.001

[bib13] Frayling TM, Timpson NJ, Weedon MN, Zeggini E, Freathy RM, Lindgren CM et al. A common variant in the FTO gene is associated with body mass index and predisposes to childhood and adult obesity. Science 2007; 316: 889–894. 17434869 10.1126/science.1141634 PMC2646098

[bib14] Comuzzie AG, Cole SA, Laston SL, Voruganti VS, Haack K, Gibbs RA et al. Novel genetic loci identified for the pathophysiology of childhood obesity in the Hispanic population. PLoS ONE 2012; 7: e51954. 23251661 10.1371/journal.pone.0051954 PMC3522587

[bib15] Manning AK, Hivert M-F, Scott RA, Grimsby J, Bouatia-Naji N, Chen H et al. A genome-wide approach accounting for body mass index identifies genetic variants influencing fasting glycemic traits and insulin resistance. Nat Genet 2012; 44: 659–669. 22581228 10.1038/ng.2274 PMC3613127

[bib16] Linder K, Wagner R, Hatziagelaki E, Ketterer C, Heni M, Machicao F et al. Allele summation of diabetes risk genes predicts impaired glucose tolerance in female and obese individuals. PLoS ONE 2012; 7: e38224. 22768041 10.1371/journal.pone.0038224 PMC3387191

[bib17] Giannini C, Dalla Man C, Groop L, Cobelli C, Zhao H, Shaw MM et al. Co-occurrence of risk alleles in or near genes modulating insulin secretion predisposes obese youth to prediabetes. Diabetes Care 2014; 37: 475–482. 24062323 10.2337/dc13-1458 PMC3898754

[bib18] Xi B, Zhao X, Shen Y, Wu L, Hou D, Cheng H et al. An obesity genetic risk score predicts risk of insulin resistance among Chinese children. Endocrine 2014; 47: 825–832. 24619288 10.1007/s12020-014-0217-y

[bib19] Stryjecki C, Peralta-Romero J, Alyass A, Karam-Araujo R, Suarez F, Gomez-Zamudio J et al. Association between PPAR-γ2 Pro12Ala genotype and insulin resistance is modified by circulating lipids in Mexican children. Sci Rep 2016; 6: 24472. 27075119 10.1038/srep24472 PMC4830984

[bib20] Sham P, Bader JS, Craig I, O’Donovan M, Owen M. DNA pooling: a tool for large-scale association studies. Nat Rev Genet 2002; 3: 862–871. 12415316 10.1038/nrg930

[bib21] Meaburn E, Butcher LM, Liu L, Fernandes C, Hansen V, Al-Chalabi A et al. Genotyping DNA pools on microarrays: tackling the QTL problem of large samples and large numbers of SNPs. BMC Genomics 2005; 6: 52. 15811185 10.1186/1471-2164-6-52 PMC1079828

[bib22] Macgregor S, Visscher PM, Montgomery G. Analysis of pooled DNA samples on high density arrays without prior knowledge of differential hybridization rates. Nucleic Acids Res 2006; 34: e55. 16627870 10.1093/nar/gkl136 PMC1440945

[bib23] Kirov G, Nikolov I, Georgieva L, Moskvina V, Owen MJ, O’Donovan MC. Pooled DNA genotyping on Affymetrix SNP genotyping arrays. BMC Genomics 2006; 7: 27. 16480507 10.1186/1471-2164-7-27 PMC1382214

[bib24] Gaj P, Maryan N, Hennig EE, Ledwon JK, Paziewska A, Majewska A et al. Pooled sample-based GWAS: a cost-effective alternative for identifying colorectal and prostate cancer risk variants in the Polish population. PLoS ONE 2012; 7: e35307. 22532847 10.1371/journal.pone.0035307 PMC3331859

[bib25] Pearson JV, Huentelman MJ, Halperin RF, Waibhav TD, Melquist S, Homer N et al. Identification of the genetic basis for complex disorders by use of pooling-based genomewide single-nucleotide-polymorphism association studies. Am J Hum Genet 2007; 80: 126–139. 17160900 10.1086/510686 PMC1785308

[bib26] Marshall WA, Tanner JM. Variations in the pattern of pubertal changes in boys. Arch Dis Child 1970; 45: 13–23. 5440182 10.1136/adc.45.239.13 PMC2020414

[bib27] Marshall WA, Tanner JM. Variations in pattern of pubertal changes in girls. Arch Dis Child 1969; 44: 291–303. 5785179 10.1136/adc.44.235.291 PMC2020314

[bib28] Pan H, Cole TJ. LMS growth a Microsoft Excel add-in to access growth references based on the LMS method. Version 2.77 [Online], 2012Available from http://www.healthforallchildren.co.uk/.

[bib29] Sacks DB, Arnold M, Bakris GL, Bruns DE, Horvath AR, Kirkman MS et al. Guidelines and recommendations for laboratory analysis in the diagnosis and management of diabetes mellitu. Clin Chem 2011; 57: e1–47. 21617152 10.1373/clinchem.2010.161596

[bib30] Matsuda M, DeFronzo RA. Insulin sensitivity indices obtained from oral glucose tolerance testing: comparison with the euglycemic insulin clamp. Diabetes Care 1999; 22: 1462–1470. 10480510 10.2337/diacare.22.9.1462

[bib31] Matthews DR, Hosker JP, Rudenski AS, Naylor BA, Treacher DF, Turner RC. Homeostasis model assessment: insulin resistance and beta-cell function from fasting plasma glucose and insulin concentrations in man. Diabetologia 1985; 28: 412–419. 3899825 10.1007/BF00280883

[bib32] Macgregor S, Zhao ZZ, Henders A, Nicholas MG, Montgomery GW, Visscher PM. Highly cost-efficient genome-wide association studies using DNA pools and dense SNP arrays. Nucleic Acids Res 2008; 36: e35. 18276640 10.1093/nar/gkm1060 PMC2346606

[bib33] Earp MA, Rahmani M, Chew K, Brooks-Wilson A. Estimates of array and pool-construction variance for planning efficient DNA-pooling genome wide association studies. BMC Med Genomics 2011; 4: 81. 22122996 10.1186/1755-8794-4-81 PMC3247851

[bib34] Faul F, Erdfelder E, Lang AG, Buchner A. G*Power 3: a flexible statistical power analysis program for the social, behavioral, and biomedical sciences. Behav Res Methods 2007; 39: 175–191. 17695343 10.3758/bf03193146

[bib35] Benjamini Y, Kriger AM, Yekutieli D. Adaptive linear step-up procedures that control the false discovery rate. Biometrika 2006; 93: 491–507.

[bib36] International HapMap Consortium. The International HapMap Project. Nature 2003; 426: 789–796. 14685227 10.1038/nature02168

[bib37] Birney E, Soranzo N. Human genomics: the end of the start for population sequencing. Nature 2015; 526: 52–53. 26432243 10.1038/526052a

[bib38] Visscher PM, Le Hellard S. Simple method to analyze SNP-based association studies using DNA pools. Genet Epidemiol 2003; 24: 291–296. 12687646 10.1002/gepi.10240

[bib39] Riaz M, Lorés-Motta L, Richardson AJ, Lu Y, Montgomery G, Omar A et al. GWAS study using DNA pooling strategy identifies association of variant rs4910623 in OR52B4 gene with anti-VEGF treatment response in age-related macular degeneration. Sci Rep 2016; 6: 37924. 27892514 10.1038/srep37924 PMC5124940

[bib40] Khong JJ, Burdon KP, Lu Y, Laurie K, Leonardos L, Baird PN et al. Pooled genome wide association detects association upstream of FCRL3 with Graves’ disease. BMC Genomics 2016; 17: 939. 27863461 10.1186/s12864-016-3276-z PMC5116198

[bib41] Craig JE, Hewitt AW, McMellon AE, Henders AK, Ma L, Wallace L et al. Rapid inexpensive genome-wide association using pooled whole blood. Genome Res 2009; 19: 2075–2080. 19801603 10.1101/gr.094680.109 PMC2775600

[bib42] Burdon KP, Macgregor S, Bykhovskaya Y, Javadiyan S, Li X, Laurie KJ et al. Association of polymorphisms in the hepatocyte growth factor gene promoter with keratoconus. Invest Ophthalmol Vis Sci 2011; 52: 8514–8519. 22003120 10.1167/iovs.11-8261 PMC3208191

[bib43] Postula M, Janicki PK, Rosiak M, Kaplon-Cieslicka A, Trzepla E, Filipiak KJ et al. New single nucleotide polymorphisms associated with differences in platelets reactivity in patients with type 2 diabetes treated with acetylsalicylic acid: genome-wide association approach and pooled DNA strategy. J Thromb Thrombolysis 2013; 36: 65–73. 23054467 10.1007/s11239-012-0823-6

[bib44] Rampersaud E, Damcott CM, Fu M, Shen H, McArdle P, Shi X et al. Identification of novel candidate genes for type 2 diabetes from a genome-wide association scan in the Old Order Amish: evidence for replication from diabetes-related quantitative traits and from independent populations. Diabetes 2007; 56: 3053–3062. 17846126 10.2337/db07-0457

[bib45] Liu F, Roth RA. Grb-IR: a SH2-domain-containing protein that binds to the insulin receptor and inhibits its function. Proc Natl Acad Sci USA 1995; 92: 10287–10291. 7479769 10.1073/pnas.92.22.10287 PMC40781

[bib46] GoDARTS and UKPDS Diabetes Pharmacogenetics Study Group Wellcome Trust Case Control Consortium. Common variants near ATM are associated with glycemic response to metformin in type 2 diabetes. Nat Genet 2011; 43: 117–120. 21186350 10.1038/ng.735 PMC3030919

[bib47] Schalch DS, McFarlin DE, Barlow MH. An unusual form of diabetes mellitus in ataxia telangiectasia. N Engl J Med 1970; 282: 1396–1402. 4192270 10.1056/NEJM197006182822503

[bib48] Miles PD, Treuner K, Latronica M, Olefsky JM, Barlow C. Impaired insulin secretion in a mouse model of ataxia telangiectasia. Am J Physiol Endocrinol Metab 2007; 293: e70–e74. 17356010 10.1152/ajpendo.00259.2006

[bib49] Cronin S, Berger S, Ding J, Schymick JC, Washecka N, Hernandez DG et al. A genome-wide association study of sporadic ALS in a homogenous Irish population. Hum Mol Genet 2008; 17: 768–774. 18057069 10.1093/hmg/ddm361

[bib50] Perurena OH, Festoff BW. Reduction in insulin receptors in amyotrophic lateral sclerosis correlates with reduced insulin sensitivity. Neurology 1987; 37: 1375–1379. 3614662 10.1212/wnl.37.8.1375

[bib51] Pradat PF, Bruneteau G, Gordon PH, Dupuis L, Bonnefont-Rousselot D, Simon D et al. Impaired glucose tolerance in patients with amyotrophic lateral sclerosis. Amyotroph Lateral Scler 2010; 11: 166–171. 20184518 10.3109/17482960902822960

[bib52] Mariosa D, Kamel F, Bellocco R, Ye W, Fang F. Association between diabetes and amyotrophic lateral sclerosis in Sweden. Eur J Neurol 2015; 22: 1436–1442. 25600257 10.1111/ene.12632 PMC4506907

[bib53] Jäger S, Wahl S, Kröger J, Sharma S, Hoffmann P, Floegel A et al. Genetic variants including markers from the exome chip and metabolite traits of type 2 diabetes. Sci Rep 2017; 7: 6037. 28729637 10.1038/s41598-017-06158-3 PMC5519666

[bib54] Macgregor S. Most pooling variation in array-based DNA pooling is attributable to array error rather than pool construction error. Eur J Hum Genet 2007; 15: 501–504. 17264871 10.1038/sj.ejhg.5201768

[bib55] Lustig RH, Mulligan K, Noworolski SM, Tai VW, Wen MJ, Erkin-Cakmak A et al. Isocaloric fructose restriction and metabolic improvement in children with obesity and metabolic syndrome. Obesity (Silver Spring) 2016; 24: 453–460. 26499447 10.1002/oby.21371 PMC4736733

[bib56] Verma S, Arikawa E, McNeill JH. Long-term endothelin receptor blockade improves cardiovascular function in diabetes. Am J Hypertens 2001; 14: 679–687. 11465653 10.1016/s0895-7061(01)01302-4

[bib57] Wood AR, Esko T, Yang J, Vedantam S, Pers TH, Gustafsson S et al. Defining the role of common variation in the genomic and biological architecture of adult human height. Nat Genet 2014; 46: 1173–1186. 25282103 10.1038/ng.3097 PMC4250049

[bib58] Stoynev N, Dimova I, Rukova B, Hadjidekova S, Nikolova D, Toncheva D et al. Gene expression in peripheral blood of patients with hypertension and patients with type 2 diabetes. J Cardiovasc Med 2014; 15: 702–709. 10.2459/JCM.0b013e32835dbcc8 23337395

[bib59] Nejentsev S, Howson JM, Walker NM, Szeszko J, Field SF, Stevens HE et al. Localization of type 1 diabetes susceptibility to the MHC class I genes HLA-B and HLA-A. Nature 2007; 450: 887–892. 18004301 10.1038/nature06406 PMC2703779

[bib60] Nakabayashi K, Tajima A, Yamamoto K, Takahashi A, Hata K, Takashima Y et al. Identification of independent risk loci for Graves’ disease within the MHC in the Japanese population. J Hum Genet 2011; 56: 772–778. 21900946 10.1038/jhg.2011.99

[bib61] Weng L, Taylor KD, Chen YD, Sopko G, Kelsey SF, Bairey Merz CN et al. Genetic loci associated with nonobstructive coronary artery disease in Caucasian women. Physiol Genomics 2016; 48: 12–20. 26534935 10.1152/physiolgenomics.00067.2015 PMC4757024

[bib62] Newgard CB, Littman DR, van Genderen C, Smith M, Fletterick RJ. Human brain glycogen phosphorylase. Cloning, sequence analysis, chromosomal mapping, tissue expression, and comparison with the human liver and muscle isozymes. J Biol Chem 1988; 263: 3850–3857. 3346228

[bib63] Kato K, Shimizu A, Kurobe N, Takashi M, Koshikawa T. Human brain-type glycogen phosphorylase: quantitative localization in human tissues determined with an immunoassay system. J Neurochem 1989; 52: 1425–1432. 2651563 10.1111/j.1471-4159.1989.tb09189.x

[bib64] Wu C, Jin X, Tsueng G, Afrasiabi C, Su AI. BioGPS: building your own mash-up of gene annotations and expression profiles. Nucleic Acids Res 2016; 44: 313–316. 10.1093/nar/gkv1104 PMC4702805 26578587

[bib65] O’Seaghdha CM, Wu H, Yang Q, Kapur K, Guessous I, Zuber AM et al. Meta-analysis of genome-wide association studies identifies six new loci for serum calcium. PLoS Genet 2013; 9. 10.1371/journal.pgen.1003796 PMC3778004 24068962

[bib66] Yasuda K, Hurukawa Y, Okuyama M, Kikuchi M, Yoshinaga K. Glucose-tolerance and insulin-secretion in patients with parathyroid disorders - effect of serum-calcium on insulin release. N Engl J Med 1975; 292: 501–504. 1117893 10.1056/NEJM197503062921003

[bib67] Sun G, Vasdev S, Martin GR, Gadag V, Zhang H. Altered calcium homeostasis is correlated with abnormalities of fasting serum glucose, insulin resistance, and beta-cell function in the Newfoundland population. Diabetes 2005; 54: 3336–3339. 16249463 10.2337/diabetes.54.11.3336

[bib68] Das A, Durrant D, Koka S, Salloum FN, Xi L, Kukreja RC. Mammalian target of rapamycin (mTOR) inhibition with rapamycin improves cardiac function in type 2 diabetic mice potential role of attenuated oxidative stress and altered contractile protein expression. J Biol Chem 2014; 289: 4145–4160. 24371138 10.1074/jbc.M113.521062 PMC3924280

[bib69] Philips KB, Kurtoglu M, Leung HJ, Liu H, Gao N, Lehrman MA et al. Increased sensitivity to glucose starvation correlates with downregulation of glycogen phosphorylase isoform PYGB in tumor cell lines resistant to 2-deoxy-D-glucose. CANCER Chemother Pharmacol 2014; 73: 349–361. 24292700 10.1007/s00280-013-2358-8 PMC4570497

