# Supplementary material for: Identification of novel alleles associated with insulin resistance in childhood obesity using pooled-DNA genome-wide association study approach
Source: Int J Obes (Lond). 2018 Feb 6;42(4):686–95. doi: 10.1038/ijo.2017.293 (PMC5984073; doi:10.1038/ijo.2017.293)
Supplement: Supplementary Material Legends [file ijo2017293x1.docx]

**Suppl. Figure 1:** Manhattans plots for top five significant hits after pooled GWAS: Chr1:21266624 (A), Chr2:102014739 (B), Chr5:142005685 (C), Chr6:30007810 (D) and Chr20:25274318. X-axis represents genomic coordinates for SNPs and Y-axis represents negative logarithm of the SNP’s association p-value.

**Supplemental Table 1 :** Normalized mean-rank values of SNPs (above threshold 1) for the IR+ vs IR- comparison. For SNPs overlapping multiple transcripts, all transcripts IDs, together with the gene-related information (gene, predicted mutation effect, gene section), are listed. Chromosomal positions are in b37 coordinates.

**Supplemental Table 2.** Pooling variances used in the statistical test as a mean of 9 comparisons of IR+ and IR- pools. The number of SNPs per pool was 495884.
