# Supplementary figures and images for: Identification of novel alleles associated with insulin resistance in childhood obesity using pooled-DNA genome-wide association study approach
Source: Int J Obes (Lond). 2018 Feb 6;42(4):686–95. doi: 10.1038/ijo.2017.293 (PMC5984073; doi:10.1038/ijo.2017.293)

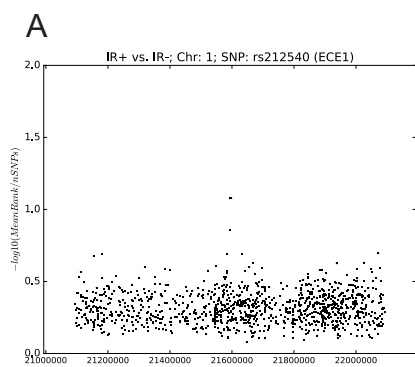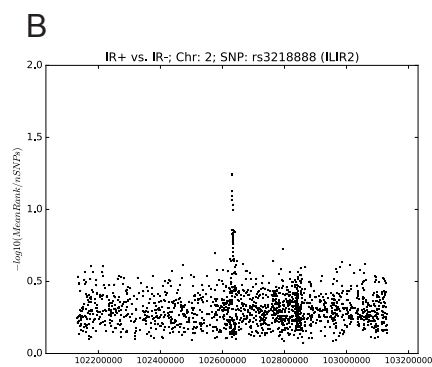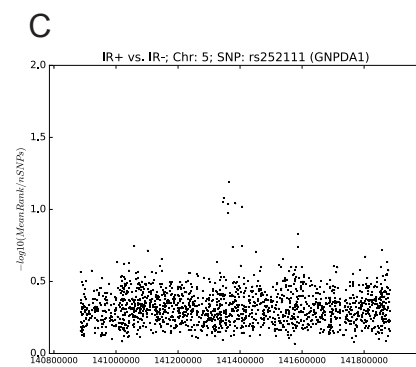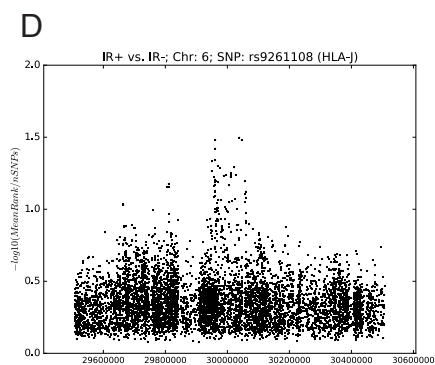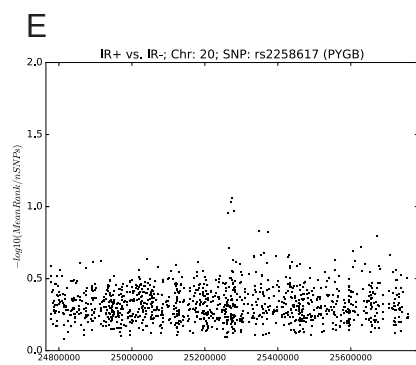

Supplement: Supplementary Figure 1 [file ijo2017293x2.pdf]
