# Supplementary material for: Identification of novel alleles associated with insulin resistance in childhood obesity using pooled-DNA genome-wide association study approach
Source: Int J Obes (Lond). 2018 Feb 6;42(4):686–95. doi: 10.1038/ijo.2017.293 (PMC5984073; doi:10.1038/ijo.2017.293)
Supplement: Supplementary Table 1 [file ijo2017293x3.docx]

**SUPPLEMENTARY TABLE 1:** Normalized mean-rank values of SNPs (above threshold 1) for the IR+ vs IR- comparison. For SNPs overlapping multiple transcripts, all transcripts IDs, together with the gene-related information (gene, predicted mutation effect, gene section), are listed. Chromosomal positions are in b37 coordinates.

| **SNP ID** | **Chr** | | **Pos** | **Score** | **Transcript(s)** | **Gene(s)** | **Mutation(s)** | **Section** |
| --- | --- | --- | --- | --- | --- | --- | --- | --- |
| **rs212540** | 1 | 21593117 | | 1.076 | NM_001113347, NM_001397, NM_001113349, NM_001113348 | *ECE1, ECE1, ECE1, ECE1* | Silent, Silent, Silent, Silent | INTRON |
| **rs212549** | 1 | 21598377 | | 1.078 | NM_001113347, NM_001397, NM_001113349, NM_001113348 | *ECE1, ECE1, ECE1, ECE1* | Silent, Silent, Silent, Silent | INTRON |
| **rs2125421** | 1 | 96458324 | | 1.098 |  |  |  |  |
| **rs234668** | 1 | 184828377 | | 1.019 | NM_052966 | *FAM129A* | Silent | INTRON |
| **rs232567** | 2 | 38394739 | | 1.080 | NR_027252 | *CYP1B1-AS1* | Silent | INTRON |
| **rs3218878** | 2 | 102629474 | | 1.065 | NM_004633, NM_173343 | *IL1R2, IL1R2* | Silent, Silent | INTRON |
| **rs3218883** | 2 | 102630273 | | 1.125 | NM_004633, NM_173343 | *IL1R2, IL1R2* | Silent, Silent | INTRON |
| **rs3218885** | 2 | 102630386 | | 1.093 | NM_004633, NM_173343 | *IL1R2, IL1R2* | Silent, Silent | INTRON |
| **rs3218888** | 2 | 102630695 | | 1.240 | NM_004633, NM_173343 | *IL1R2, IL1R2* | Silent, Silent | INTRON |
| **rs3218892** | 2 | 102631201 | | 1.245 | NM_004633, NM_173343 | *IL1R2, IL1R2* | Silent, Silent | INTRON |
| **rs2236926** | 2 | 102635048 | | 1.034 | NM_004633, NM_173343 | *IL1R2, IL1R2* | Silent, Silent | INTRON |
| **rs280060** | 4 | 94958025 | | 1.019 |  |  |  |  |
| **rs280058** | 4 | 94961303 | | 1.107 |  |  |  |  |
| **rs4699837** | 4 | 96170439 | | 1.039 | NM_003728 | *UNC5C* | Silent | INTRON |
| **rs4699847** | 4 | 96220207 | | 1.096 | NM_003728 | *UNC5C* | Silent | INTRON |
| **rs477451** | 4 | 105528877 | | 1.193 |  |  |  |  |
| **rs475106** | 4 | 177656790 | | 1.157 | NM_005429 | *VEGFC* | Silent | INTRON |
| **rs248074** | 5 | 126504182 | | 1.031 |  |  |  |  |
| **rs248093** | 5 | 126512372 | | 1.130 |  |  |  |  |
| **rs248103** | 5 | 126518136 | | 1.008 |  |  |  |  |
| **rs6596007** | 5 | 130588550 | | 1.175 |  |  |  |  |
| **rs6596010** | 5 | 130625411 | | 1.360 | NM_001038702, NM_020240 | *CDC42SE2, CDC42SE2* | Silent, Silent | INTRON |
| **rs6596013** | 5 | 130651969 | | 1.505 | NM_001038702, NM_020240 | *CDC42SE2, CDC42SE2* | Silent, Silent | INTRON |
| **rs6596020** | 5 | 130749955 | | 1.067 |  |  |  |  |
| **rs2522052** | 5 | 131798487 | | 1.001 | NR_045116 | *C5orf56* | Silent | INTRON |
| **rs252139** | 5 | 141345218 | | 1.052 |  |  |  |  |
| **rs252137** | 5 | 141346830 | | 1.081 | NM_183399, NM_001201365 | *RNF14, RNF14* | Silent, Silent | INTRON |
| **rs252096** | 5 | 141362272 | | 1.037 | NM_183399, NM_001201365, NM_004290, NM_183400, NM_183398, NM_183401 | *RNF14, RNF14, RNF14, RNF14, RNF14, RNF14* | Silent, Silent, Silent, Silent, Silent, Silent | INTRON |
| **rs252101** | 5 | 141364752 | | 1.189 | NM_183399, NM_001201365, NM_004290, NM_183400, NM_183398, NM_183401 | *RNF14, RNF14, RNF14, RNF14, RNF14, RNF14* | Silent, Silent, Silent, Silent, Silent, Silent | INTRON |
| **rs252111** | 5 | 141385250 | | 1.041 | NM_005471 | *GNPDA1* | Silent | INTRON |
| **rs252128** | 5 | 141406997 | | 1.020 |  |  |  |  |
| **rs252155** | 5 | 150065544 | | 1.026 |  |  |  |  |
| **rs9368558** | 6 | 28133306 | | 1.003 |  |  |  |  |
| **rs1233604** | 6 | 28734676 | | 1.078 |  |  |  |  |
| **rs1233608** | 6 | 28736189 | | 1.111 |  |  |  |  |
| **rs1233609** | 6 | 28736201 | | 1.002 |  |  |  |  |
| **rs1233615** | 6 | 28739584 | | 1.004 |  |  |  |  |
| **rs1233616** | 6 | 28739884 | | 1.035 |  |  |  |  |
| **rs209152** | 6 | 28846462 | | 1.013 |  |  |  |  |
| **rs209151** | 6 | 28846724 | | 1.058 |  |  |  |  |
| **rs3129085** | 6 | 29664637 | | 1.028 |  |  |  |  |
| **rs3129084** | 6 | 29664664 | | 1.038 |  |  |  |  |
| **rs2523780** | 6 | 29806331 | | 1.155 |  |  |  |  |
| **rs2523778** | 6 | 29807860 | | 1.157 |  |  |  |  |
| **rs2523773** | 6 | 29810113 | | 1.157 |  |  |  |  |
| **rs2523772** | 6 | 29810325 | | 1.177 |  |  |  |  |
| **rs9260919** | 6 | 29948884 | | 1.266 |  |  |  |  |
| **rs9260923** | 6 | 29949889 | | 1.332 |  |  |  |  |
| **rs9260931** | 6 | 29957508 | | 1.222 |  |  |  |  |
| **rs9260933** | 6 | 29957866 | | 1.164 |  |  |  |  |
| **rs9260934** | 6 | 29957982 | | 1.087 |  |  |  |  |
| **rs9260937** | 6 | 29958526 | | 1.090 |  |  |  |  |
| **rs9260946** | 6 | 29959254 | | 1.129 |  |  |  |  |
| **rs9260951** | 6 | 29959538 | | 1.123 |  |  |  |  |
| **rs9260953** | 6 | 29959830 | | 1.172 |  |  |  |  |
| **rs9260954** | 6 | 29959935 | | 1.187 |  |  |  |  |
| **rs9260955** | 6 | 29960037 | | 1.206 |  |  |  |  |
| **rs9260957** | 6 | 29960083 | | 1.202 |  |  |  |  |
| **rs9260959** | 6 | 29960870 | | 1.340 |  |  |  |  |
| **rs9260963** | 6 | 29961071 | | 1.422 |  |  |  |  |
| **rs9260968** | 6 | 29961439 | | 1.479 |  |  |  |  |
| **rs9260973** | 6 | 29961580 | | 1.195 |  |  |  |  |
| **rs9260998** | 6 | 29963622 | | 1.196 |  |  |  |  |
| **rs9261041** | 6 | 29966718 | | 1.042 |  |  |  |  |
| **rs9261043** | 6 | 29966726 | | 1.042 |  |  |  |  |
| **rs9261045** | 6 | 29966867 | | 1.009 |  |  |  |  |
| **rs9261080** | 6 | 29968987 | | 1.012 | NR_026751 | *ZNRD1-AS1* | Silent | EXON |
| **rs9261093** | 6 | 29970685 | | 1.299 | NR_026751 | *ZNRD1-AS1* | Silent | INTRON |
| **rs9261095** | 6 | 29971014 | | 1.066 | NR_026751 | *ZNRD1-AS1* | Silent | INTRON |
| **rs9261096** | 6 | 29971039 | | 1.187 | NR_026751 | *ZNRD1-AS1* | Silent | INTRON |
| **rs9261108** | 6 | 29975587 | | 1.289 | NR_026751, NR_024240 | *ZNRD1-AS1, HLA-J* | Silent, Silent | INTRON, EXON |
| **rs9261130** | 6 | 29980445 | | 1.064 | NR_026751 | *ZNRD1-AS1* | Silent | INTRON, EXON |
| **rs9261151** | 6 | 29987738 | | 1.233 | NR_026751 | *ZNRD1-AS1* | Silent | INTRON, EXON |
| **rs9261156** | 6 | 29991538 | | 1.016 | NR_026751 | *ZNRD1-AS1* | Silent | INTRON, EXON |
| **rs9261171** | 6 | 29996121 | | 1.002 | NR_026751 | *ZNRD1-AS1* | Silent | INTRON, EXON |
| **rs9261174** | 6 | 29996855 | | 1.237 | NR_026751 | *ZNRD1-AS1* | Silent | INTRON, EXON |
| **rs9261203** | 6 | 30005043 | | 1.165 | NR_026751 | *ZNRD1-AS1* | Silent | INTRON |
| **rs9261207** | 6 | 30006482 | | 1.048 | NR_026751 | *ZNRD1-AS1* | Silent | INTRON |
| **rs9261216** | 6 | 30010139 | | 1.225 | NR_026751 | *ZNRD1-AS1* | Silent | INTRON |
| **rs9261218** | 6 | 30011275 | | 1.249 | NR_026751 | *ZNRD1-AS1* | Silent | INTRON |
| **rs9261219** | 6 | 30011451 | | 1.236 | NR_026751 | *ZNRD1-AS1* | Silent | INTRON |
| **rs9261224** | 6 | 30013887 | | 1.057 | NR_026751 | *ZNRD1-AS1* | Silent | INTRON |
| **rs9261257** | 6 | 30022425 | | 1.290 | NR_026751 | *ZNRD1-AS1* | Silent | INTRON |
| **rs9261261** | 6 | 30024365 | | 1.070 | NR_026751 | *ZNRD1-AS1* | Silent | INTRON |
| **rs9261265** | 6 | 30026350 | | 1.239 | NR_026751 | *ZNRD1-AS1* | Silent | INTRON |
| **rs9261269** | 6 | 30030114 | | 1.003 | NM_170783, NM_014596 | *ZNRD1, ZNRD1* | Silent, Silent | INTRON |
| **rs9261291** | 6 | 30038712 | | 1.498 | NM_170769, NM_025236 | *RNF39, RNF39* | Silent, Silent | UTR |
| **rs9261307** | 6 | 30045846 | | 1.481 |  |  |  |  |
| **rs9261309** | 6 | 30045958 | | 1.480 |  |  |  |  |
| **rs9261360** | 6 | 30055667 | | 1.193 |  |  |  |  |
| **rs9261361** | 6 | 30055933 | | 1.052 |  |  |  |  |
| **rs9261365** | 6 | 30057675 | | 1.106 |  |  |  |  |
| **rs9261370** | 6 | 30058956 | | 1.123 |  |  |  |  |
| **rs9261372** | 6 | 30059055 | | 1.081 |  |  |  |  |
| **rs9276820** | 6 | 32829276 | | 1.008 |  |  |  |  |
| **rs9276827** | 6 | 32830904 | | 1.048 |  |  |  |  |
| **rs9276842** | 6 | 32839628 | | 1.048 |  |  |  |  |
| **rs9276847** | 6 | 32840384 | | 1.093 |  |  |  |  |
| **rs9276859** | 6 | 32841544 | | 1.085 |  |  |  |  |
| **rs9276863** | 6 | 32843043 | | 1.070 |  |  |  |  |
| **rs9276881** | 6 | 32844214 | | 1.061 |  |  |  |  |
| **rs9276899** | 6 | 32848168 | | 1.058 |  |  |  |  |
| **rs240153** | 6 | 101067135 | | 1.030 | NM_006828 | *ASCC3* | Silent | INTRON |
| **rs240149** | 6 | 101073730 | | 1.018 | NM_006828 | *ASCC3* | Silent | INTRON |
| **rs2237447** | 7 | 50707844 | | 1.014 | NM_001001550, NM_001001555, NM_001001549, NM_005311 | *GRB10, GRB10, GRB10, GRB10* | Silent, Silent, Silent, Silent | INTRON |
| **rs1228913** | 7 | 84128927 | | 1.140 |  |  |  |  |
| **rs1228897** | 7 | 84145460 | | 1.120 |  |  |  |  |
| **rs41939** | 7 | 117950678 | | 1.290 |  |  |  |  |
| **rs41948** | 7 | 117963926 | | 1.303 |  |  |  |  |
| **rs41955** | 7 | 117970074 | | 1.364 |  |  |  |  |
| **rs41960** | 7 | 117974788 | | 1.092 |  |  |  |  |
| **rs2189125** | 7 | 118007075 | | 1.020 |  |  |  |  |
| **rs327967** | 9 | 108001107 | | 1.134 |  |  |  |  |
| **rs327960** | 9 | 108008159 | | 1.152 | NM_080546 | *SLC44A1* | Silent | INTRON |
| **rs10819101** | 9 | 128768663 | | 1.067 |  |  |  |  |
| **rs304500** | 10 | 91149563 | | 1.056 |  |  |  |  |
| **rs227070** | 11 | 108211412 | | 1.011 | NM_000051 | *ATM* | Silent | INTRON |
| **kgp11290060** | 12 | 116414606 | | 1.084 | NM_015335 | *MED13L* | Silent | INTRON |
| **kgp4333196** | 17 | 66562710 | | 1.098 | NM_001243746, NM_017565 | *FAM20A, FAM20A* | Silent, Silent | INTRON, EXON |
| **kgp432664** | 17 | 66677575 | | 1.031 |  |  |  |  |
| **kgp4331782** | 17 | 76485703 | | 1.162 | NM_173628 | *DNAH17* | Silent | INTRON |
| **rs4789670** | 17 | 80151805 | | 1.008 | NM_198082 | *CCDC57* | Silent | INTRON |
| **rs2258135** | 20 | 25272323 | | 1.031 | NM_002862 | *PYGB* | Silent | INTRON |
| **rs2258617** | 20 | 25274318 | | 1.060 | NM_002862 | *PYGB* | Silent | INTRON |
| **kgp1925073** | 21 | 35974498 | | 1.031 | NM_004414, NM_203417 | *RCAN1, RCAN1* | Silent, Silent | INTRON |
| **kgp1931258** | 21 | 42333908 | | 1.118 |  |  |  |  |
| **kgp24649877** | 22 | 42349051 | | 1.022 | NR_024355 | *BK250D10.8* | Silent | INTRON |
| **kgp24649098** | 22 | 49857370 | | 1.067 |  |  |  |  |
| **rs4826709** | X | 5964744 | | 1.058 | NM_181332, NM_020742 | *NLGN4X, NLGN4X* | Silent, Silent | INTRON |
| **rs4828540** | X | 16883491 | | 1.117 | NM_002893, NM_001198719 | *RBBP7, RBBP7* | Silent, Silent | INTRON |
| **rs4828547** | X | 17121235 | | 1.114 | NM_004726, NM_001080975 | *REPS2, REPS2* | Silent, Silent | INTRON |
| **rs808567** | X | 32598518 | | 1.176 | NM_004006, NM_004010, NM_004007, NM_000109, NM_004009 | *DMD, DMD, DMD, DMD, DMD* | Silent, Silent, Silent, Silent, Silent | INTRON |
| **rs5963539** | X | 37311627 | | 1.040 | NM_000950, NM_001142395, NM_001173489, NM_001173490 | *PRRG1, PRRG1, PRRG1, PRRG1* | Silent, Silent, Silent, Silent | INTRON |
| **rs3002418** | X | 39707666 | | 1.029 |  |  |  |  |
| **rs3002401** | X | 39743294 | | 1.016 |  |  |  |  |
| **rs6614540** | X | 50308735 | | 1.023 |  |  |  |  |
| **rs6614541** | X | 50311693 | | 1.006 |  |  |  |  |
| **rs3000841** | X | 58317986 | | 1.015 |  |  |  |  |
| **rs321014** | X | 77620061 | | 1.204 |  |  |  |  |
| **rs321013** | X | 77621340 | | 1.169 |  |  |  |  |
| **rs321011** | X | 77622606 | | 1.226 |  |  |  |  |
| **rs321009** | X | 77623623 | | 1.134 |  |  |  |  |
| **rs321034** | X | 77635122 | | 1.166 |  |  |  |  |
| **rs321053** | X | 77671115 | | 1.197 |  |  |  |  |
| **rs321054** | X | 77671395 | | 1.212 |  |  |  |  |
| **rs321061** | X | 77677099 | | 1.177 |  |  |  |  |
| **rs321068** | X | 77685925 | | 1.136 |  |  |  |  |
| **rs5944795** | X | 101203646 | | 1.073 |  |  |  |  |
| **rs5944854** | X | 101377997 | | 1.016 |  |  |  |  |
| **rs6622141** | X | 106309583 | | 1.019 | NM_001171080, NM_018301 | *RBM41, RBM41* | Silent, Silent | INTRON |
| **rs6622143** | X | 106313508 | | 1.718 | NM_001171080, NM_018301 | *RBM41, RBM41* | Silent, Silent | INTRON |
| **rs6622145** | X | 106322625 | | 1.898 | NM_001171080, NM_018301 | *RBM41, RBM41* | Silent, Silent | INTRON |
| **rs6622152** | X | 106344058 | | 1.871 | NM_001171080, NM_018301 | *RBM41, RBM41* | Silent, Silent | INTRON |
| **rs5962787** | X | 106362675 | | 1.248 |  |  |  |  |
| **rs6622169** | X | 106406361 | | 1.839 | NM_017681, NR_033676 | *NUP62CL, NUP62CL* | Silent, Silent | INTRON |
| **rs6622171** | X | 106414433 | | 1.751 | NM_017681, NR_033676 | *NUP62CL, NUP62CL* | Silent, Silent | INTRON |
| **rs1285573** | X | 106415810 | | 1.029 | NM_017681, NR_033676 | *NUP62CL, NUP62CL* | Silent, Silent | INTRON |
| **rs6622172** | X | 106422864 | | 1.754 | NM_017681, NR_033676 | *NUP62CL, NUP62CL* | Silent, Silent | INTRON |
| **rs5962794** | X | 106423926 | | 1.436 | NM_017681, NR_033676 | *NUP62CL, NUP62CL* | Silent, Silent | INTRON |
| **rs6622185** | X | 106469429 | | 1.730 | NM_001169154, NM_173494 | *CXorf41, CXorf41* | Silent, Silent | INTRON |
| **rs6622188** | X | 106491179 | | 1.531 |  |  |  |  |
| **rs6622207** | X | 106530363 | | 1.127 |  |  |  |  |
| **rs3001972** | X | 119017269 | | 1.043 |  |  |  |  |
| **rs5907701** | X | 139938600 | | 1.071 |  |  |  |  |
| **rs5907713** | X | 139960461 | | 1.161 |  |  |  |  |
| **rs5907721** | X | 139985284 | | 1.022 |  |  |  |  |
