# Supplementary material for: Identification of novel alleles associated with insulin resistance in childhood obesity using pooled-DNA genome-wide association study approach
Source: Int J Obes (Lond). 2018 Feb 6;42(4):686–95. doi: 10.1038/ijo.2017.293 (PMC5984073; doi:10.1038/ijo.2017.293)
Supplement: Supplementary Table 2 [file ijo2017293x4.docx]

**Supplemental Table 2.** Pooling variances used in the statistical test as a mean of 9 comparisons of IR+ and IR- pools. The number of SNPs per pool was 495884.

| **IR pools compared** | **Mean pooling variances** |
| --- | --- |
| IR+ pool1 : IR- pool1 | 0.000782 |
| IR+ pool1 : IR- pool2 | 0.000887 |
| IR+ pool1 : IR- pool3 | 0.000971 |
| IR+ pool2 : IR- pool1 | 0.000814 |
| IR+ pool2 : IR- pool2 | 0.000894 |
| IR+ pool2 : IR- pool3 | 0.000925 |
| IR+ pool3 : IR- pool1 | 0.000772 |
| IR+ pool3 : IR- pool2 | 0.000828 |
| IR+ pool3 : IR- pool3 | 0.000877 |
